# The NAC Transcription Factor Gene *OsY37* (ONAC011) Promotes Leaf Senescence and Accelerates Heading Time in Rice

**DOI:** 10.3390/ijms18102165

**Published:** 2017-10-17

**Authors:** Yousra El Mannai, Kenta Akabane, Keiichiro Hiratsu, Namiko Satoh-Nagasawa, Hiroetsu Wabiko

**Affiliations:** 1 Faculty of Bioresource Sciences, Department of Biological Production, Akita Prefectural University, Akita 010-0195, Japan; yousra@akita-pu.ac.jp (Y.E.M.); kenta.redwing@gmail.com (K.A.); satohnagasawa@akita-pu.ac.jp (N.S.-N.); 2 Department of Applied Chemistry, National Defense Academy of Japan, Yokosuka 239-8686, Japan; khiratsu@nda.ac.jp

**Keywords:** leaf senescence, chlorophyll degradation, NAC (*NAM*, *ATAF*, and *CUC*) gene, RNAi (RNA interference), CRES-T (Chimeric Repressor Silencing Technology), heading, grain

## Abstract

Leaf senescence is an important physiological process involving the degradation of a number of metabolites and their remobilization to new reproductive and storage organs. NAC (*NAM*, *ATAF*, and *CUC*) transcription factors are reported as important regulators of the senescence process. Here, we describe the identification and functional characterization of the NAC transcription factor gene, *OsY37* (*Oryza sativa* Yellow37, ONAC011) obtained from *Oryza sativa* cv. *indica*, and *japonica*. We created transgenic plants expressing the *OsY37* gene under the control of a strong and constitutive CaMV35S promoter. The resulting transgenic plants overexpressing *OsY37* gene showed early heading and precocious senescence phenotype of flag leaves compared with wild-type plants. By contrast, blocking the function of this gene via RNAi (RNA interference) and CRES-T (Chimeric Repressor Silencing Technology) technology, delayed both heading time and leaf senescence. Furthermore, knockdown of *OsY37* expression caused dwarfism and high accumulation of chlorophyll during the vegetative phase. Irrespective of early or delayed senescence, transgenic plants showed reduced grain yields. Our results indicate that *OsY37* acts as a positive regulator of heading and senescence during the reproductive phase in rice. In addition, *OsY37* may be involved in plant development and grain yield.

## 1. Introduction

Plants undergo distinct developmental phases during their life cycle; mainly vegetative, reproductive, ripening, and the senescence phase. Senescence is the final stage of development in plants during which metabolic and morphological changes occur, leading to the death of the whole plant. During leaf senescence, chlorophyll degradation takes place in the earliest stages [1], and proteins and nucleic acids are degraded by newly synthesized proteases [2,3] and nucleases [4,5] respectively. The resulting catabolic products are mobilized from leaves for use in the growing parts of the plant or to be stored in developing seeds. Therefore, senescence is characterized by the transition from nutrient assimilation to nutrient remobilization [6,7,8,9]. In particular, flag leaves surviving close to the end of senescence, may provide carbon source to sink organs and play a major role in grain yield compared to the other leaves [10]. Therefore, natural leaf senescence is recognized not only as a negative, aging process but also as involving active biochemical and physiological events. All these events have strong effects on the relocation of nutrients and as a consequence, on grain quality in wheat [11] and crop yield in rice [12] as well as in other crop plants [13]. In rice plants, flowering immediately follows heading and these two processes precede leaf senescence progress. Flowering and senescence are strictly defined serial events and intimately associated. For example, *Arabidopsis ELF3* (early-flowering 3) is a regulator of floral induction [14] and its homolog *OsELF3.1* promotes leaf senescence in rice [15]. Furthermore, natural senescence is also closely associated to oxidative and abiotic stresses. Hydrogen peroxide triggers production of reactive oxygen species, which induce the expression of a number of senescence associated genes [16], and furthermore the induced senescence promotes accumulation of hydrogen peroxide as a positive feedback mechanism [17].

Comprehensive analysis for metabolic, physiological, and genetic alterations during leaf senescence and its close relationship to senescence-associated gene (SAG) expression have been extensively studied [16,18,19,20,21,22]. Alteration of gene expression profile is primarily controlled by transcription factors. NAC (*NAM*, *ATAF*, and *CUC*) transcription factor genes were originally discovered as morphological determinants in plants. For example, the *nam* (*no apical meristem*) mutant of petunia is unable to develop a shoot apical meristem [23], and double mutations in *cuc1* and *cuc2* (*cup-shaped cotyledon*) of *Arabidopsis* cause cup-shaped cotyledons [24]. NAC family genes encode the polypeptides consisting of a highly conserved N-terminal half segment (NAC domain) and a unique C-terminal half region. The NAC domain contains a nuclear localization signal and a characteristic DNA-binding domain, whereas the C-terminal motif is considered to determine the specificity of the target genes and hence to participate in diverse aspects of physiological functions. Based on the subdomain configurations, NAC family genes are classified into several classes [25,26,27]. It was later found that NAC genes regulate positively and negatively the expression of a number of SAGs. Additionally, previous studies demonstrated that overexpression of *OsNAP*, *OsNAC2* from rice and *NAC016* from *Arabidopsis* accelerates leaf senescence, whereas lowered expression of these genes in mutants or via RNAi (RNA interference) inhibition results in delayed senescence [12,28,29]. Similarly, *Arabidopsis ANAC032* [17] and *ORS1* (*PRE1 SISTER1*) a paralog of *ORE1* (*ORESARA1*)/*ANAC092* [30] promote leaf senescence in *Arabidopsis*. In contrast, similar experiments indicate that the NAC genes, *JUB1* (*JUNGBRUNNEN1*) and *VNI2* (*VND-INTERACTING2*) of *Arabidopsis* delay and hence suppress senescence [31,32].

Here we report that the expression of the novel NAC gene of rice (ONAC011, Os06g0675600), which we designated *OsY37*^I^ (*Oryza sativa* Yellow37) from *indica* rice and *OsY37*^N^ from *japonica* rice, is induced during natural leaf senescence. Furthermore, transgenic rice plants overexpressing the *OsY37* gene showed promoted heading and natural senescence. In contrast, knockdown of the gene delayed heading and senescence and also affected normal vegetative growth and development.

## 2. Results

### 2.1. Isolation of the OsY37^I^ Gene

To identify suitable leaf materials for isolation of SAGs from rice, we first measured the chlorophyll content of the flag leaf, second and third leaves, from the top, after the heading date. These leaves were excised, extracted, and chlorophyll was measured using a spectrometer. Chlorophyll contents decreased gradually starting from the heading date to reach their lowest value 60 days later. At 34 days after heading, the chlorophyll content was reduced to 50% of the initial concentration in the flag leaf and second leaf. At this time point, equal amounts of these leaves were mixed and used for RNA isolation. The first and second green leaves from the top were harvested 10 days before heading and used as control. Total RNA was subjected to differential display using random forward 10-mer primers and an oligo-dT reverse primer. One forward primer (CAGGCCCTTC) preferentially amplified a fragment of 251 bp from senescent leaves compared with green leaves (Figures S1 and S2). Other amplified fragments were similarly amplified in both tissues, but the intensity of some fragments was larger in green leaves compared with senescent leaves. The full-length clone of the 251 bp-segment was obtained from the cDNA library from *indica* rice, and sequence analysis revealed that the gene corresponds to the NAC family gene ONAC011 (Os06g0675600) from *japonica* rice [25]. Thus, we designated the cloned genes as *OsY37*^I^ and *OsY37*^N^ from *japonica* respectively. Both coding regions showed identical nucleotide sequences except that a 9 bp stretch is absent in *OsY37*^I^ and three nucleotide positions are different between the two genes. *OsY37*^I^ and *OsY37*^N^ encode polypeptides comprising 301 and 304 amino acid (aa) residues, respectively, and they differ from each other in 2 aa residues as a result of base substitution (Figure S2). Based on the close resemblance in gene structure between *OsY37*^I^ and *OsY37*^N^, functional characteristics and regulatory mechanisms of the two genes are believed to be almost identical to each other.

### 2.2. Increased Accumulation of OsY37 Transcripts during Natural Senescence

**Figure 1:**
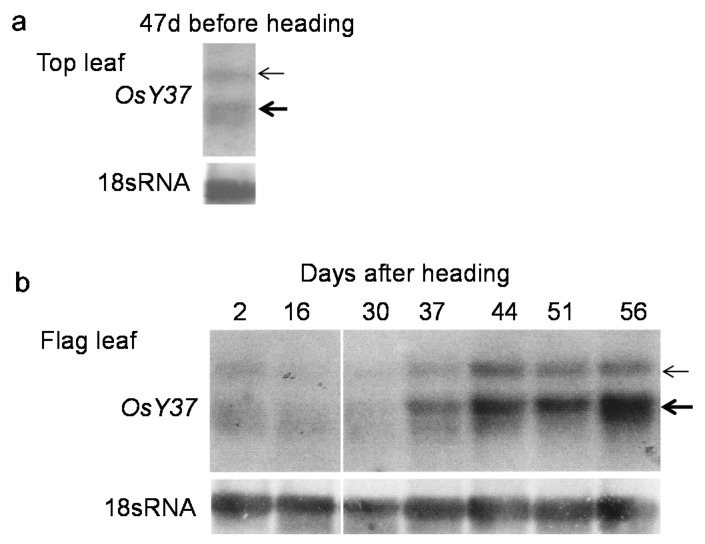
Northern hybridization analysis of *OsY37* gene transcript accumulation during leaf senescence. Rice cv. “Akitakomachi” top leaves were harvested at 47 d before heading (**a**) and flag leaves after heading dates (**b**). Total RNA was extracted from the leaves of several plants at the same growth stages. RNA (20 µg) was separated by agarose/formaldehyde gel electrophoresis, blotted onto a nylon membrane, and allowed to hybridize with the [α-^32^P]-dCTP-labeled 251 bp *OsY37*^I^ segment (Figure S2). Thick and thin arrows point to 1 and 3–4 kb transcripts, respectively. The same membrane was re-hybridized with cDNA of *Arabidopsis thaliana* 18S rRNA as a loading control, which is shown below the *OsY37*^I^ segment-probed hybridization. After hybridization, the membrane was washed under high stringency washing conditions.

Age-dependent increase of *OsY37* transcript levels in the flag leaf was determined by Northern hybridization analysis. We used, as a probe, a 251 bp segment comprising the 3′-end of the gene with a unique sequence among the NAC family genes (Figure S2). Basal levels of transcripts of 1 kb and 3–4 kb were observed in young green top leaves at 47 days before heading (Figure 1a). Comparable transcript levels of both sizes accumulated as long as 23–30 days after heading (Figure 1b), indicating that the basal levels of *OsY37* transcripts accumulated irrespective of developmental stage. Moreover, genomic information indicates that the total length of the genomic *OsY37* comprises 4 kb, including three exons separated by two introns, whereas matured mRNA extended 906 bp in length (Figure S2).

Both of the 1 and 3–4 kb transcripts, started to accumulate to increased levels at 37 days after heading (Figure 1b). Accumulation of *OsY37* transcripts increased during the following developmental stages to attain their maximum levels at 56 days corresponding to the mid-senescence phase (Figure 1b).

### 2.3. Induction of OsY37 Expression Under Nitrogen Starvation Conditions

**Figure 2:**
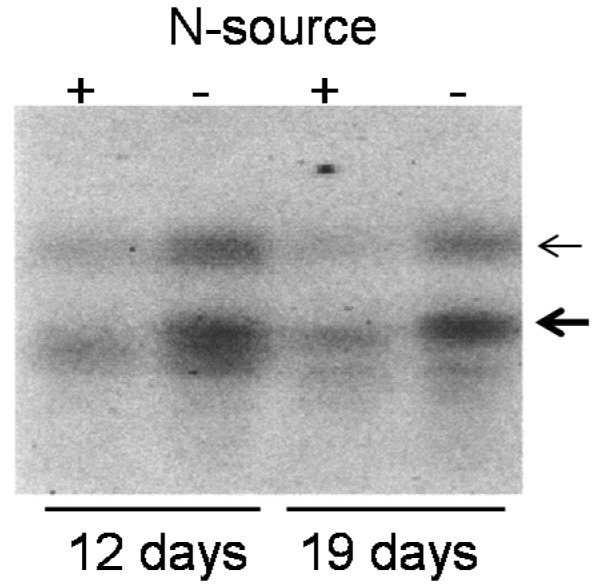
Induction of *OsY37* expression under nitrogen starvation conditions. Rice cv. “Akitakomachi” seeds were sown on soil and grown to the juvenile seedling stage. Whole seedlings were transferred to pots containing vermiculite with (+) or without (−) a nitrogen source. After the indicated number of days, RNA (100 μg) was extracted and Northern hybridization analysis was performed as described in Figure 1. Thick and thin arrows correspond to 1 and 3–4 kb transcripts, respectively.

Application of nitrogen fertilizer is crucial to obtain appropriate crop yields and hence limitation of the nitrogen source promotes leaf senescence [33]. Considering the possible involvement of the *OsY37* gene in this process, we determined *OsY37* transcript accumulation upon nitrogen starvation. Rice seedlings were grown in natural soil containing complete nutrients, and then transferred to hypotonic growth conditions with or without a nitrogen source. Total RNA was isolated and subjected to Northern hybridization analysis. *OsY37* transcripts of both 1 and 3–4 kb sizes were highly accumulated at 12 and 19 days after nitrogen starvation, whereas the transcript levels remained low when a nitrogen source was fully supplied (Figure 2).

### 2.4. Nuclear Localization of GFP/OsY37 Fusion Polypeptide

**Figure 3:**
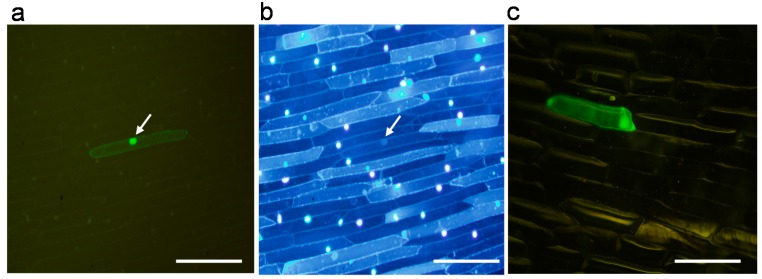
Nuclear localization of GFP/OsY37^I^ fusion protein in onion epidermal cells. The *GFP/OsY37*^I^ gene (**a**,**b**) and *GFP* (**c**) constructs under the control of the CaMV35S promoter (clone pGFOSY) were introduced into onion epidermal cells by particle bombardment. Intracellular localization of the GFP protein after 24 h was examined by fluorescence microscopy (**a**,**c**). Nuclei were stained with 4′,6-diamidino-2-phenylindole dihydrochloride (DAPI) (**b**). Arrows indicate the nucleus where accumulation of GFP was observed. Bar = 500 μm

To substantiate the relevance of *OsY37* as a transcriptional regulator, we constructed the chimeric gene fusion *GFP*/*OsY37*^I^ under the control of the CaMV35S promoter, and then examined whether the fusion polypeptide is localized in the nucleus of plant cells. The chimeric construct, pGFOSY containing CaMV35S::*GFP*:*OsY37*^I^ fusion as well as psGFP(S65T) containing CaMV35S::*GFP* constructs were introduced into onion epidermal cells by particle bombardment. The fluorescence signal of GFP:OsY37^I^ polypeptide was localized exclusively in the nucleus (Figure 3a,b), whereas the signal of GFP alone was distributed throughout the cell including the nucleus (Figure 3c).

### 2.5. Effects of Overexpression and Knockdown of OsY37 on Heading Date and Leaf Senescence

**Figure 4:**
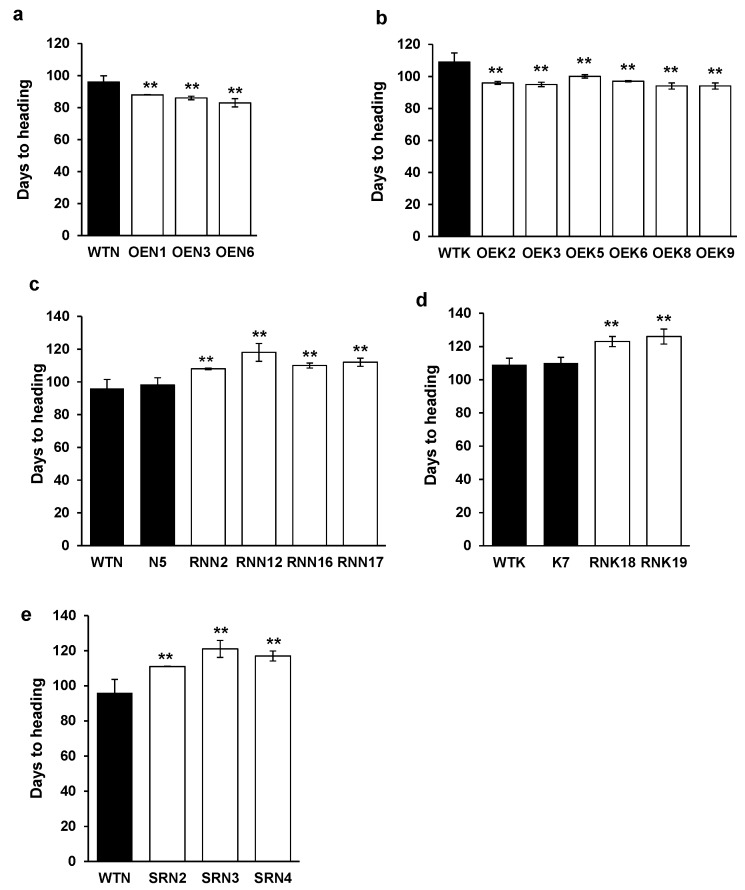
Number of days to heading. Seeds were sown in soil and allowed to grow in a greenhouse under natural photoperiod conditions. Time required from sowing to heading was monitored. Triplicate panicles from single plants were investigated (Student’s *t*-test, ** *p* < 0.01). Heading was followed by flowering in average of 2 days (1–3 days). Overexpressing (OE) lines contain CaMV35S::*OsY37*^I^ construct and repressing (RE) RNAi lines contain CaMV35S::RNAi-*OsY37*^I^ construct, and SRD(SUPRD)X lines contain *OsY37*^I^ promoter::*O*s*Y37*^N^-SRDX construct. Wild-type Nipponbare (WTN) and Nipponbare OE lines (OEN) (**a**); wild-type Kinmaze (WTK) and Kinmaze OE lines (OEK) (**b**); WTN and Nipponbare RNAi lines (RNN) (**c**); WTK and Kinmaze RNAi lines (RNK) (**d**); WTN and Nipponbare SRDX lines (SRN) (**e**).

To explore the physiological function of the *OsY37* gene in regulating leaf senescence in rice, we created a chimeric construct in which the *OsY37* gene expression is driven by a strong, constitutive CaMV35S promoter and transferred the construct to rice cv. Nipponbare and Kinmaze plants. The resulting transgenic plants were overexpressing (OE) the *OsY37* gene. The number of days from sowing to heading of wild-type plants ranged from 95 to 97 days for Nipponbare while it ranged from 108 to 110 days for Kinmaze (Figure 4). We employed the two rice cultivars since they exhibit different heading dates, therefore effects of the overexpression could be better observed. Indeed, after 2–3 days from heading, rice plants set flowers in both cultivars and senescence symptoms started upon flowering. The OE transgenic plants grew normally without noticeable phenotypic alterations during the vegetative growth phase. The transgenic lines of both cultivars, however, showed a significant reduction in the number of days to heading compared with wild-type plants. Nipponbare OE lines started heading 5–12 days earlier, while in Kinmaze OE lines, heading occurred 9–13 days earlier than in the respective wild-type (Figure 4a,b).

**Figure 5:**
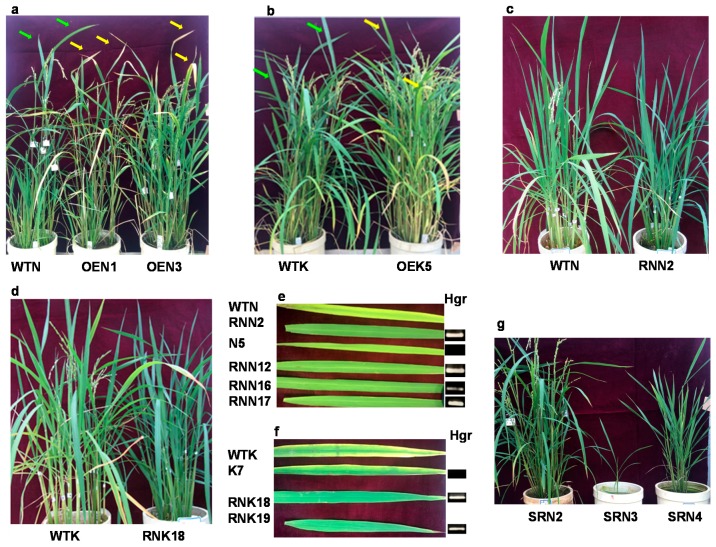
Phenotype of rice plants during senescence. Wild-type and OE lines of Nipponbare (**a**) and Kinmaze (**b**) at 20 days after heading of wild-type Nipponbare and Kinmaze plants. Green arrows indicate wild-type flag leaves showing greenish in color, while yellow arrows depict OE lines with yellowing color. Wild-type and RNAi lines of Nipponbare (**c**) and Kinmaze (**d**) at 15 days after heading of wild-type. Color development of flag leaves of RNAi lines at 70 days after heading of wild-type plants (**e**). Lines N5 (Nipponbare) and K7 (Kinmaze) are Hgr^−^ segregates from the primary T0 transformants of RNN5 and RNK7 respectively. Absence of transgene was verified by PCR (polymerase chain reaction) of genomic DNA by using Hgr-specific primers, hph1 and hph2 (**e**,**f**, Table 1). SRDX lines of Nipponbare at 20 days after heading of wild-type (**g**). Names of plant lines are shown in Figure 4.

Later, OE lines showed an early leaf senescence compared with wild-type plants. Visual inspection of the plants revealed that early senescence is most clearly recognized in the flag leaves (Figure 5a,b). The yellowing color of the flag leaves started from the distal end and progressively extended proximally with time.

To examine the progress of the senescence process in more detail, we determined the profile of the chlorophyll content in flag leaves for wild-type and transgenic rice plants upon heading.

**Figure 6:**
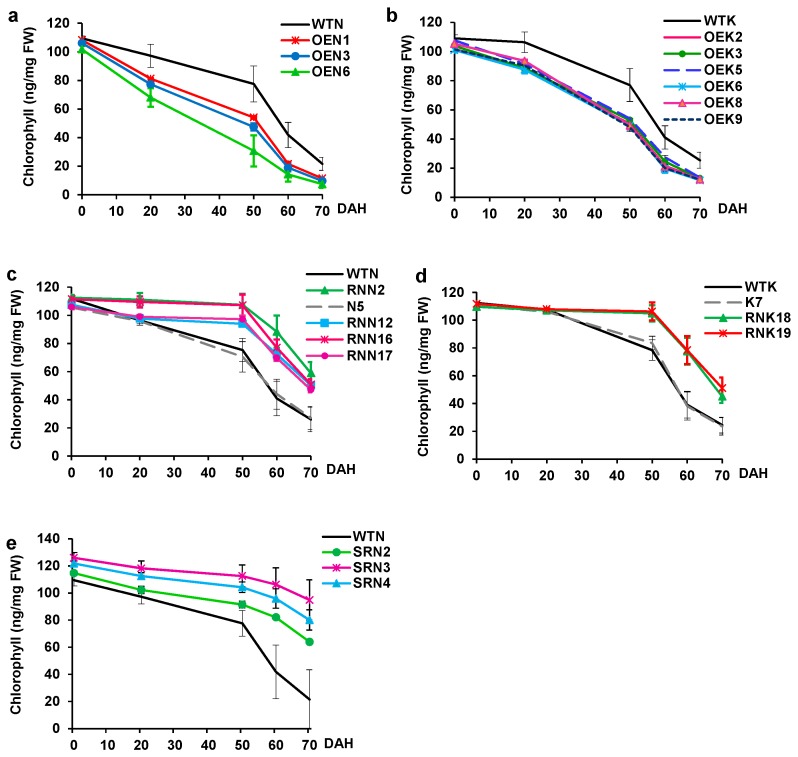
Chlorophyll contents in the flag leaves after heading. Central portions of flag leaves of wild-type and transgenic-lines were excised and the chlorophyll was extracted at the indicated days after heading (DAH). Triplicated samples were analyzed from single plants. Mean values with standard deviations are shown. Wild-type, OE lines of Nipponbare (**a**) and Kinmaze (**b**), RNAi lines of Nipponbare (**c**) and Kinmaze (**d**), and SRDX lines of Nipponbare (**e**). Names of plant lines are shown in Figure 4.

Chlorophyll contents decreased in the wild-type and the pattern of declining chlorophyll shows a biphasic profile (Figure 6). At the first stage, chlorophyll levels started to decrease immediately in Nipponbare and 20 days after heading in Kinmaze respectively, but the rate of decline is gradual in both plants (Figure 6). In contrast, at the second stage (30–40 days after heading), chlorophyll contents started to decrease rapidly to reach their lowest levels at the end of this stage (Figure 6).

Our results also showed that chlorophyll levels were comparable among transgenic and wild-type plants at heading time. However, chlorophyll degradation of OE lines started at an early stage compared with wild-type (Figure 6a,b). Indeed, at 20 days after heading, chlorophyll content was reduced by 10% to 20% in OE plants of both Nipponbare and Kinmaze compared with wild-type. Consequently, at 50 days after heading, 50% of chlorophyll content was degraded in most of the OE plants, whereas it was maintained at 80% for wild-type. Induced senescence by the gene overexpression continued to proceed and was manifested by continuous chlorophyll degradation until the end of this process (Figure 6a,b). Our results further show that early senescence phenotypes exhibited by the OE plants are not merely the consequence of the early heading. Indeed, significant difference was observed between the rate of chlorophyll degradation and that predicted from the differential heading date between wild-type and OE plants.

**Figure 7:**
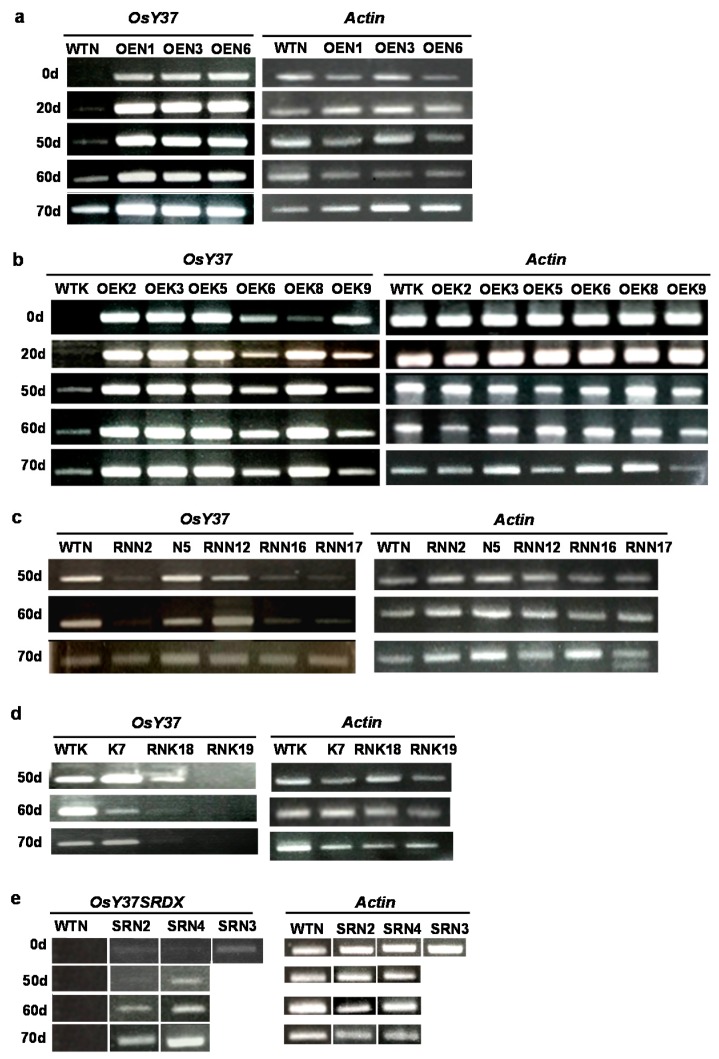
Transcriptional gene expression determined by reverse transcription polymerase chain reaction (RT-PCR) analysis. Total RNA was extracted from flag leaves at the indicated days after heading. Semi-quantititative RT-PCR was performed using *OsY37* primers Osy2 and Osy752 which give rise to the amplification of 754 DNA fragment (**a**–**d**). To amplify *OsY37*SRDX transcript, *OsY37-*specific forward primer; OsyPri1 and SRDX-specific reverse primer; SRDX-R were used*.* Actin gene expression analysis was carried out similarly with forward; OsActinF318 and reverse; OsActinR675 primers for internal standard. Wild-type and OE lines of Nipponbare (**a**); Kinmaze (**b**); RNAi lines of Nipponbare (**c**); Kinmaze (**d**); and SRDX lines of Nipponbare (**e**). Names of plant lines are shown in Figure 4.

Gene expression of the OE lines was investigated by semi-quantitative reverse transcription polymerase chain reaction (Clontech) analysis. In the case of wild-type Kinmaze and Nipponbare, transcript accumulation was non-detectable at the heading date, but increased gradually with time to reach the maximum levels at 70 days when chlorophyll had declined to the lowest levels (Figure 7a,b). In contrast, transgenic OE plants showed high accumulation of transcripts even at 0 day and remained constant or slightly increased (the Kinmaze line, OEK8) throughout the senescence periods (Figure 7a,b). These results indicate that the promoted senescence as well as the heading time of the OE transgenic plants are the consequence of stably increased expression of *OsY37*.

We next investigated physiological alteration caused by repressing the internal *OsY37* gene expression by introducing CaMV35S::*OsY37*^I^/RNAi construct to rice. Small interfering RNAs generated from RNAi construct knockdown the endogenous target transcript levels. We additionally examined for the hygromycin-sensitive (Hgr^−^) segregates derived from the primary transformed plants of the T1 generation. The RNAi lines from both cultivars showed slightly reduced growth rate during the vegetative phase (Figure 6c,d). When they reached maturation, all the RNAi lines showed delayed heading by 12–22 days compared with respective wild-type plants (Figure 4c,d). Senescence of RNAi lines is delayed by approximately 30 days compared with wild-type. RNAi lines exhibited a dark green color phenotype (Figure 5c–f). By contrast, flag leaves of wild-type and Hgr^−^ segregates lines, N5 (Nipponbare) and K7 (Kinmaze), exhibited yellow color at an early stage after heading (Figure 5e,f). Correspondingly, for both rice cultivars, chlorophyll contents started to decline 50 days after heading in RNAi lines, whereas they started decline immediately for wild-type Nipponbare and 20 days after heading for wild-type Kinmaze (Figure 6c,d).

RT-PCR analysis revealed that accumulation of *OsY37* transcripts varied among plants. At the interval of 50–70 days after heading, characterized by significant differential chlorophyll content between wild-type and transgenic rice plants, wild-type and Hgr^−^ segregates of Nipponbare showed almost equal levels of transcript accumulation. By contrast, the RNAi transgenic lines except for RNN12, showed no or substantially less transcript levels (Figure 7c). The RNN12 line showed only slightly less amounts of transcripts but comparable to wild-type levels at 60 days. At the end of the senescence process, 70 days after heading, the transcripts were uniformly accumulated among all plants (Figure 7b). This result can be explained by the reduction of the creation of small fragment derivatives from RNAi at the latest stages of senescence.

Similarly, for Kinmaze, transgenic lines displayed low accumulation level of *OsY37* transcripts in line RNK18, and no accumulation in line RNK19. At 60 days, transcripts were reduced in both lines and did not resume even at 70 days. However, in wild-type and Hgr^−^ segregates (K7) transcripts were continuously present from 50 to 70 days (Figure 7d). These results indicate RNAi construct effectively suppressed intragenic *OsY37* transcript accumulation and that interference of *OsY37* gene expression is responsible for the delay of heading time and leaf senescence in rice. 

To confirm the results of knockdown of the *OsY37* gene expression using RNAi, we employed the CRES-T method. In this technology, the repression polypeptide domain termed SRDX(SUPRD)X (where X denotes any amino acid) is fused to the C-terminal end of the transcription factor. Upon the introduction of this fused construct to plants, the SRDX-tagged polypeptide suppresses dominantly and specifically the corresponding intragenic transcriptional activity [34].

We created the SRDX construct where *OsY37*^N^-SRDX is regulated under the control of its own promoter and transferred it to Nipponbare, aiming at low expression levels of the chimeric gene during the vegetative phase and activation during senescence. Hence, endogenous *OsY37* activity is specifically controlled irrespective of the developmental stages of the plants. The transgenic plants exhibited varying degrees of dwarf phenotype. In particular, SRN3 line showed extremely defective growth with only 40% height of wild-type plant at full maturity. In addition, this line developed a single tiller and a single panicle with a limited number of spikelets (Figure 5g). Heading date of the SRDX lines was delayed by 15–25 days compared with wild-type (Figure 4e and Figure 5g). At the heading date, transgenic plants maintained a level of chlorophyll 5–15% higher than wild-type, resulting in dark green leaf phenotype as described above. Subsequently, all the transgenic lines showed a reduced rate of chlorophyll degradation profile. Indeed, for wild-type, the chlorophyll content declined to the lowest level 70 days after heading, while transgenic plants exhibited stay-green phenotype and maintained chlorophyll at 55% and 75% in SRN2 and SRN3 at 70 days respectively (Figure 6e).

*OsY37*-SRDX transcripts were determined by RT-PCR using the *OsY37*-specific forward primer (OsyRri1) and SRDX-specific reverse primer (SRDX-R). Basal transcript levels were recognized at the onset of heading (0 day) in both lines SRN2 and SRN4 and slightly higher levels in SRN3 (Figure 7e). The transcript levels increased gradually over time in a senescence-dependent manner in the lines SRN2 and SRN4 (Figure 7e). This increment reflects that OsY37-SRDX is regulated under the control of *OsY37* promoter which is activated during senescence. RT-PCR for the SRN3 line was performed at 0 day only since this dwarf plant developed a single small flag leaf used for RNA preparation at this time. Nonetheless, *OsY37* expression levels after the heading time correlated well with the extent of delayed senescence.

### 2.6. Effects of Modulated OsY37 Versions on Vegetative Growth and Plant Development

To investigate the involvement of *OsY37* in plant growth and development, we created SRDX-tagged *OsY37*^I^ under the control of strong and constitutive promoters, CaMV35S and Act1. Subsequently, we attempted to introduce the resulting chimeric gene to rice plants through *Agrobacterium*-mediated gene transfer. *Agrobacterium* harboring the chimeric gene was infected to embryonic unorganized rice callus, selected for Hgr^R^. The resulting callus was transferred to regeneration medium. Although Hgr^R^ callus were successfully obtained, the regeneration of plants from the callus to shoots failed. In contrast, 10–20% regenerated transgenic plants were consistently obtained upon transformation by the vector alone. We performed the same transformation experiments three times; however no regeneration of shoot was obtained. These results indicate that the repressor version of the OsY37 protein, under the control of CaMV35S and Act1 in rice cells interferes with the normal regeneration process. In addition, we transferred these constructs to *Arabidopsis thaliana* to observe phenotypic alteration. All of the resulting transgenic seedlings showed dwarfing phenotype with less than 80% in height compared with wild-type plants (Figure S3). These results are a first indication that *OsY37* might be involved in normal growth and development of rice plants in addition to controlling the senescence process.

### 2.7. Effects of Modified OsY37 Genes on Grain Yield in Rice

**Figure 8:**
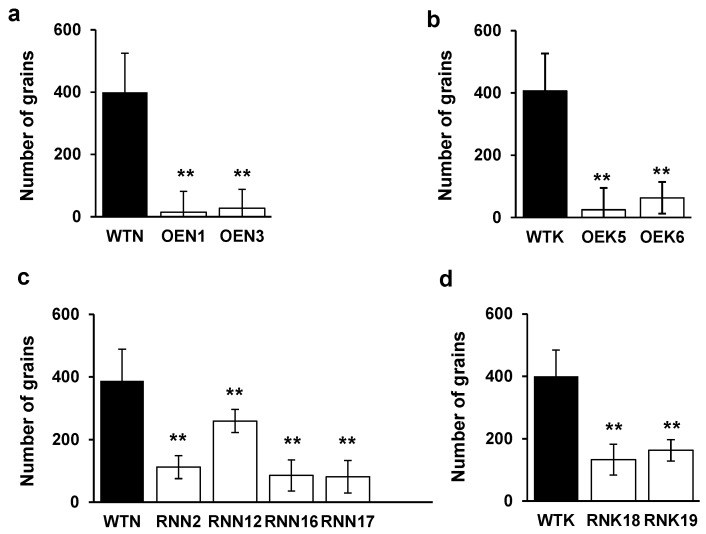
Number of grains in wild-type, OE, and RNAi transgenic lines. Three progenies obtained from the individual transgenic lines were sown and allowed to reach maturity. Matured grains from individual plants were harvested and total grain number was counted. Mean values of grain number from triplicate samples are shown (Student’s *t*-test, ** *p* < 0.01). OE lines of Nipponbare (**a**); Kinmaze (**b**); RNAi lines of Nipponbare (**c**); Kinmaze (**d**). Names of plant lines are shown in Figure 4.

Since overexpressing and repressing versions of *OsY37* affected heading time and senescence progression, grain yields are susceptible to be affected as a result. Mature rice grains were harvested and counted. Compared with wild-type, OE lines showed reduced yield by 85–93% and RNAi lines showed 35–75% reduction. Thus, we found that both OE and RNAi lines showed reduced grain yields of different degrees (Figure 8). For SRDX lines, grain yield was severely affected, so that only a few seeds were harvested. These results show that the precocious senescence of flag leaves caused an incomplete grain-filling stage for OE lines. In contrast, for RNAi plants the suppression of *OsY37* generated a reduced number of tiller compared with wild type resulting in less grain yield per plant (Figure 5c,d).

## 3. Discussion

### 3.1. OsY37 Gene Expression Profile

Northern hybridization analysis using 3′ end side of the *OsY37* gene segment as a probe showed two distinct transcripts; 1 kb mRNA and an additional large 3–4 kb transcript. The 3–4 kb transcript most likely represents the precursor molecules of matured *OsY37* transcript, since the used probe is highly specific to *OsY37* transcript. Additionally, the size of the recognized transcript corresponds to the predicted size based on genome organization of the *OsY37* gene. Both transcripts accumulated at basal levels in vegetative, healthy green leaves as well as in leaves undergoing early senescence. Accumulation of transcripts was highly induced during subsequent progression of senescence. This *OsY37* gene expression profile is consistent with the existing database RiceXpro (http://ricexpro.dna.affrc.go.jp/), in which the OsNAC011 is preferentially expressed in the leaf blade during the grain ripening period, vegetative and reproductive phase of roots, the anthers, and ovary, whereas this expression is limited in vegetative leaves.

As we discuss below, *OsY37* expression promotes heading and hence flowering. Since these events occur before the senescence phenotype is observed, basal levels of the transcripts appear to be required to mediate signals leading to heading. By contrast, high transcript levels are associated with chlorophyll degradation and therefore, responsible for the induction of the irreversible senescence phase [33].

Expression of *OsY37* gene was also promoted by nitrogen starvation. This transcriptional response is consistent with a previous finding that nitrogen limitation induces senescence in *Arabidopsis* [35] and sunflower [33]. Based on the observation of expression profile of the *OsY37*, we conclude that this gene possesses dual functions; to incorporate or mediate internal triggers towards heading/flowering and to promote senescence.

### 3.2. Dual Functions of the OsY37 Gene in the Reproductive Phase

In the OE lines, the vegetative phase was restricted and transition to the reproductive phase occurred utmost 2 weeks earlier than for wild-type. Hence OE lines showed early heading/flowering. Precocious leaf senescence followed heading/flowering as evidenced by the immediate chlorophyll degradation upon heading. Conversely, heading/flowering time and senescence were significantly delayed in RNAi and SRDX lines. Consistent with these phenotypic alterations, *OsY37* gene expression levels are constantly high in OE lines. In contrast, intragenic *OsY37* transcripts were maintained at basal levels by RNAi construct. However, comparable levels of transcripts were observed at 70 days after heading in wild-type and RNAi lines of Nipponbare, while in Kinmaze RNAi lines, no transcripts were observed. Nonetheless, the resumption of *OsY37* transcript accumulation in Nipponbare RNAi lines did not affect the results of senescence since this resumption occurred after establishment of the senescence process.

Taken together, our results suggest that *OsY37* positively regulates both heading/flowering and senescence in rice. It has been reported that the other NAC genes, rice *OsNAC2* [12] and *Arabodpsis ANAC046* [36] are positive regulators for chlorophyll catabolic genes and hence promote leaf senescence. Although whether the *OsY37* gene is involved in direct catabolism of chlorophyll remains to be determined, the *OsY37* gene expression levels are associated remarkably with chlorophyll degradation. Overall, our study shows that the *OsY37* gene product can be involved in the modulation of common or mutually independent signaling pathways leading to heading, chlorophyll degradation, and senescence in rice.

Little literature describes molecular mechanisms underlying the linkage between flowering and leaf senescence. However, it is known that stress-induced senescence is closely linked to reactive oxygen species (ROS) [37] and that the NAC gene *ANAC032* mediates ROS-induced senescence [17]. Further, circadian clock regulates ROS homeostasis in *Arabidopsis* [38]. These results suggest that physiological transition is related to ROS status. Furthermore, ascorbic acid, which is a scavenger of ROS, promotes both flowering and senescence [39]. Further study is necessary to determine whether ROS involved in both flowering time and senescence are regulated by the *OsY37* gene in rice.

Similar to wild-type, heading/flowering precedes leaf senescence in transgenic plants. However, modulated *OsY37* gene expression affected both processes in transgenic plants. This result suggests that additional factors are required to primarily determine these two events and that the *OsY37* gene can promote the activities of these putative factors.

Finally, both overexpressing and repressing *OsY37* versions showed reduced grain yield compared with wild-type. In OE lines, the seed sink-strength may have not yet been established due to restricted vegetative phase. Furthermore, OE lines showed severely damaged flag leaves resulting from an early senescence observed immediately after heading. Hence the source strength of flag leaves to seeds may be weakened, due to deprivation of nutrient remobilization, the key step for grain filling. In agreement with these findings, it was reported that flag leaf contributed to 45% of rice grain yield and, when removed, was the major component for yield loss [10]. Contrary to OE versions, RNAi, and SRDX repressing versions prolonged the vegetative phase compared with the wild type plants. Although RNAi lines showed reduced yield, the grain yield of OE lines was more severely affected than RNAi lines. Reduced yield is most likely due to limited tiller number compared with wild-type. Furthermore, few grains were obtained from SRDX lines, where the number of tillers was severely affected, resulting in reduced number of panicles as exemplified by the fact that only a single panicle emerged in the SRN3 line. In addition, Vergara et al. showed that rice plants with a duration time of the vegetative phase longer than the critical duration time produced the same number of panicles, but formed fewer spikelets per panicle compared with wild-type plants [40].

In contrast to our findings, RNAi lines of *OsNAC2* and *OsNAP* delayed senescence in rice and produced high yield via modulation of abscisic acid metabolism [12,41]. It is not known, however, if these genes also affect vegetative growth and heading time. Appropriate physiological states such as duration time of the vegetative phase, can be differentially regulated by the *OsNAC2*, *OsNAP*, and *OsY37* genes, which are influential in determining the final grain yield. Since the time required for seed maturation remained unchanged irrespective of OE or RE *OsY37* versions, coordination of grain filling and senescence seems to be an important factor to obtain full grain yield.

### 3.3. Involvement of OsY37 in Vegetative Growth and Development

Our results showed that the *OsY37* gene is possibly involved in vegetative growth. Many arguments support our hypothesis. Initially, the *OsY37*-SRDX construct caused dwarfism when it was transferred to *Arabidopsis.* Although all transformations were successful, regeneration to shoots in *OsY37*-SRDX with a strong promoter failed in rice. We cannot strictly exclude that transformation of the *OsY37*-SRDX construct to rice requires different growth conditions to be accomplished successfully. Second, the *OsY37* promoter-driven *OsY37*^N^-SRDX generated dwarfing plants with a limited number of tillers. Therefore, repression of the *OsY37* gene activities via CRES-T prevents normal growth and development during the vegetative phase. Furthermore, leaves of the SRDX lines showed a dark green phenotype even at heading time, suggesting that knockdown of the gene expression prevented chlorophyll degradation during the vegetative phase. Therefore, it appears that the *OsY37* gene is involved in chlorophyll degradation not only during senescence but also during the vegetative phase.

### 3.4. Functional Comparison with the Other NAC Genes

To date, senescence associated NAC genes comprise both types; promoting senescence (*ANAC032* [17], *ANAC046* [37], *AtNAP/ANAC029* [42], *ORE1/ANAC092* [43], *ORS1/ANAC059* [30], *OsNAP* [41], *OsNAC2* [12], *ONAC106* [44]) and delaying senescence (*JUB1/ANAC042* [31], *VNI2/ANAC083* [32]). Among these genes, *ANAC032*, *ANAC046* from *Arabidopsis* and *OsNAP* and *OsNAC2* from rice positively regulate chlorophyll catabolism genes. Since *OsY37* promotes chlorophyll degradation, the gene can be classified in this group of NAC genes. Among these genes, *ANAC032* as well as *OsNAP* expression is inducible by the phytohormone abscisic acid (ABA), suggesting that these genes promote senescence via ABA signaling pathway. In contrast, *ANAC046* is not inducible by ABA despite the senescence promoting characteristics of the gene. Therefore, it is suggested that triggering senescence is not limited to a single pathway. Further, *VNI2/ANAC083* shows ABA responsive expression but delays senescence. Moreover, oxidative stress is shown to promote senescence [45,46]. It was reported that nitrogen starvation in *Arabidopsis* roots induces accumulation of H_2_O_2_, a reactive oxygen species [47]. Our results show that *OsY37*expression was induced upon nitrogen starvation. On one hand, both *ORS1/ANAC059* and *JUB1* = *ANAC042* are responsive to H_2_O_2_, a reactive oxygen species, but on the other hand, *ORS1* gene promotes senescence, whereas *JUB1* delays it. Therefore, some genes are apparently involved in common signaling pathways, but phenotypic consequence is not limited to a single type. Considering that *OsY37* expression was induced upon nitrogen starvation and that some NAC genes regulate senescence via ABA and ROS pathway, we conclude that *OsY37* can be involved in a similar senescence regulation pathway. Nevertheless, further analysis is required to identify the *OsY37* specific pathway.

## 4. Materials and Methods

### 4.1. Plant Materials and Growth Conditions

Wild-type rice (*Oryza sativa*) subsp. *japonica* cv. “Akitakomachi”, “Nipponbare” and “Kinmaze” were grown in the paddy field of Akita Prefectural University from April to October. For the laboratory work, wild-type and transgenic rice plants were grown in a greenhouse under 16 h light/8 h dark cycle at 28 and 25 °C respectively.

### 4.2. Plasmid Vectors and Primers

The pGEM-T (Promega, Madison, WI, USA) and pGEM-T Easy vectors were used to clone PCR-amplified DNA segments. We used the psGFP (S65T) expression vector which carries a modified green fluorescent protein (GFP) gene *sGFP* (S65T) under the control of the cauliflower mosaic virus 35S promoter; CaMV35S [48]. To clone the suppressing version of *OsY37*, we used the plasmids pActSRDXG and p35SSRDXG carrying rice actin, Act1 promoter, and CaMV35S promoter respectively together with the transcription factor repression domain; SRDX comprising amino acid residues, GLDLDLELRLGFA. The *japonica OsY37*^N^ full-length clone in the vector pME18SFL3, was obtained from the National Institute of Agrobiological Sciences (http://www.dna.affrc.go.jp/jp/). Versatile plant binary vector, pIG121Hm, confers hygromycin resistance (Hgr^R^) to rice plants and *Agrobacterium tumefaciens* [49] and kanamycin resistance to *A. tumefaciens* and *Escherichia coli*. Primers used in the present report are listed in Table 1.

### 4.3. Measurement of Chlorophyll Content

Leaves were excised from rice plants at different time interval after heading. Leaves were cut into pieces and were weighed immediately. The middle pieces weighing 100 mg were ground to a fine powder with liquid nitrogen and then used for extraction and measurement of chlorophyll content. Extracts were made from the leaf tissue by adding 1 mL extraction buffer composed of 0.4 M sucrose, 50 mM Tricine-KOH, 10 mM NaCl, 2 mM MgCl_2_, 0.1% (*w*/*v*) bovine serum albumin, and 0.1% (*w*/*v*) polyvinylpyrrolidone at pH 7.8, and placed on ice. Acetone was added to the samples to give a concentration of 80% (*v*/*v*) and the solution was centrifuged at 800× *g* for 10 min at 4 °C. The supernatant was saved and absorbance at 663 nm (*A*_663_) and 645 nm (*A*_645_) was measured with a spectrophotometer (Gene Spec III, Hitachi Genetic Systems, Alameda, CA, USA). Chlorophyll content (µg mL^−1^) was calculated using the formula 8.05 × *A*_663_ + 20.29 × *A*_645_ [50].

### 4.4. RNA Extraction and Northern Hybridization Analysis

Rice leaves (1 g) were ground to a fine powder in liquid nitrogen and immersed in 5 mL extraction buffer (50 mM Tris-HCl pH 8, 300 mM NaCl, 5 mM EDTA (ethylenediaminetetraacetic acid), 2% (*w*/*v*) SDS (sodium dodecyl sulfate), 10 mM β-mercaptoethanol and 1 mM aurin tricarboxylic acid). Total RNA was precipitated by LiCl [51]. Afterward, total RNA (40 µg) samples were separated by 1% agarose/formaldehyde gel electrophoresis and then blotted onto Hybond-N^+^ nylon membranes (Amersham/GE Healthcare, Chicago, IL, USA). Northern hybridization was carried out in a rapid hybridization buffer (Amersham). Membranes were washed under high stringency washing conditions in 0.1× SSC (standard saline citrate) and 0.1% SDS at 65 °C. A 251 bp *OsY37* fragment from the pGEM200 vector was radiolabeled with [α-^32^P]-dCTP (50 µCi) and used as a probe. Alternatively, for RT-PCR analysis, RNA was extracted from 100mg of green or senescing leaves using RNeasy Plant Mini Kit (QIAGEN, Hilden, Germany), then quantified fluorometrically using Qubit RNA BR Assay Kit (ThermoFisher Scientific, Waltham, MA, USA).

### 4.5. Differential Display

To identify genes differentially expressed in green leaves and naturally senescent leaves, we used differential-display reverse transcription kit (Sawady Technology, Tokyo, Japan). The upstream primers used were 10-mer oligonucleotides with an arbitrary sequence (Operon Technologies Inc., Alameda, CA, USA) and the downstream primers were 11-oligo-dT nucleotides with an additional two degenerate nucleotides at the 3′ end. Total RNA (1 µg) isolated from young, green and yellow leaves were subjected to reverse transcription to generate cDNA using the downstream primers. Subsequent PCR amplification of the cDNA using the upstream and downstream primers was performed in the presence of [α-^35^S]-dATP (3.7 × 10^4^ Bq µL^−1^) under the condition of 40 cycles of 30 s at 94 °C, 90 s at 40 °C, and 60 s at 72 °C, and one cycle of 1 min at 72 °C. The amplified radiolabeled DNA was separated by 5% polyacrylamide/8 M urea sequencing gel electrophoresis, followed by autoradiography. The portion of the gel containing the fragments showing higher intensity in senescent leaves than in green leaves was excised, immersed in 400 µL of 10 mM Tricine (pH 9.5), 0.2 mM EDTA and incubated at 94 °C for 5 min to dissolve the corresponding fragments. Aliquots were used for re-amplification of the fragment using the same primers as in the first PCR. The final product (251 bp length) was cloned into the pGEM-T vector to generate pGEM200.

### 4.6. Full-Length cDNA Library Screening and Sub-Cloning

A Rice 5′-STRETCH cDNA library was purchased from Clontech Laboratories (Palo Alto, CA, USA). The library carried total full-length cDNA and was constructed from etiolated seedlings of rice (*O. sativa* subsp. *indica* “IR36”) and cloned at the *Eco*RI restriction site of the λgt11 vector. Phages were propagated in *Escherichia coli* Y1090r^−^ cells. Approximately 10^6^ phage plaques were screened for the presence of the appropriate cDNA using, as a probe, the 251 bp senescence-specific fragment obtained from pGEM200 [52]. To clone the full-length cDNA into a plasmid vector, DNA was prepared from the λgt11 recombinant phage carrying the full-length segment, and was further amplified by PCR using the forward primer IndFw situated 20 bp upstream of the initiation codon, and the reverse primer IndRv situated 102 bp downstream of the termination codon and extended immediately upstream of a polyA sequence. The amplified segment was cloned into the pGEM-T vector to generate pGE37 (Figure S4).

### 4.7. Generation of Recombinant Clones

To generate a chimeric construct encoding the GFP/OsY37^I^ fusion polypeptide, the full-length *OsY37*^I^ cDNA was cloned into the vector plasmid sGFP (S65T). The vector contains the unique *Bsr*G and *Not*I restriction sites situated at the 3′-end of the GFP coding region and 7 bp downstream of the *Bsr*G site, respectively. *OsY37*^I^ in pGE37 was amplified using the forward primer FwOsGFP and the M13 universal RV primer. The FwOsGFP primer contained the *Bsr*GI (TGTACA) restriction site and the ATG initiation codon located 3 bp downstream of the *Bsr*G site. The amplified fragment was subjected to double digestion with *Bsr*G and *Not*I (derived from the vector), and ligated to the expression vector that had been digested with *Bsr*G and *Not*I, thus generating the clone pGFOsY. This clone harbored the OsY37^I^ polypeptide, which was immediately fused to the C-terminal end of the intact GFP polypeptide.

To obtain overexpression construct for *OsY37*^I^, the full-length was cloned in the plant vector, pIG121Hm. In pGE37 plasmid, the restriction sites; *Sph*I and *Sac*I are closely located upstream and downstream of the full-length coding region respectively (Figure S4). The pGE37^I^ was digested with *Sph*I, blunt-ended by the T4 DNA polymerase and partially digested with *Sac*I to avoid additional cut at the *Sac*I site, occurring in the intragenic region of the *OsY37*^I^. The digests were separated by agarose gel electrophoresis and the appropriate segment was recovered from the gel. The plant vector pIG121Hm was digested with *Xba*I, filled-in by the Klenow fragment, and finally digested with *Sac*I. The resulting vector was ligated to the above-mentioned *OsY37*^I^ segment generating the clone, pIG20 where the gene is regulated under the control of CaMV35S promoter and NOS (nopaline synthase) terminator.

The construction process for repressing version against intragenic *OsY37*^I^ gene activity, RNAi and CRES-T clones is diagramed in the supplemental figures. To construct RNAi*-OsY37*^I^, the full-length clone pGE37 was cut with a unique site, *Tth*111I in the coding region (position 487 from the start codon, Figure S4), filled-in by the Klenow fragment and further digested with *Sph*I. The resulting fragment comprises 3′-end side of pGE37 together with the vector (Figure S4, segment #1). Subsequently, the pGE37 was amplified by PCR with primers, forward Fw24 and reverse Rv847 to generate the segment #2 (Figure S4). The Fw24 primer is situated at 5′ flanking sequence of the *OsY37*^I^ and the Rv869 primer is located at position 847 and possesses additionally introduced *Sph*I recognition sequence. The resulting fragment was digested with *Sac*I, blunt-ended by T4 DNA polymerase, and then digested with *Sph*I (Figure S4) segment #3 and ligated to the above-mentioned segment #1 to generate pRNAiOsY37. In this clone, two regions corresponding to C-terminal domain are situated in mutually reversed orientation and hence give rise to functional sequence for RNAi inhibition for intragenic *OsY37*. To clone into a plant vector, the pRNAiOsY37DNA was digested with *Sph*I, blunt-ended by T4 DNA polymerase, then digested with *Sac*I and ligated to pIG121Hm, previously digested with *Xba*I, filled-in by Klenow, and lastly digested with *Sac*I. In this final clone, pRNAiOsY37, RNAi version of *OsY37*^I^ was regulated under the control of CaMV35S promoter (Figure S4).

The SRDX-tagged *OsY3*^I^ gene was driven either under the control of CaMV35S promoter or its own promoter. To this end, the full-length *OsY37*^I^ gene segment was amplified with the forward primer OsYFw and the reverse primer OsYRv, filled-in with T4 DNA polymerase and ligated to the *Sma*I restriction site of the pActSRDXG and p35SSRDXG vectors to generate pPAct37SRDXG and pP35S37SRDXG, respectively (Figure S5). These constructs were transferred to the plant vector pBIG-Hgr, conferring Hgr^R^ in plants, using the Gateway vector conversion system [53] (Invitrogen, Carlsbad, CA, USA) to produce pBIGAct37SRDXG and pBIG35S37SRDXG (Figure S4).

To obtain the SRDX version under the control of the *OsY37* gene, promoter region of *OsY37*^I^ was isolated from the genomic library constructed in λ phage vector (Clontech) using the coding region as a probe. Insert DNA from the identified phage was digested with *Sal*I and further subcloned in the *Sal*I site of pUC19, generating the clone, pProm17 (Figure S6). Hence we obtained 3.8 kp fragment including 169 bp coding region with the initiation codon of *OsY37*^I^. Upstream of the initiation codon, only small open reading frames with no more than 103 amino acid residues aside from *OsY37*^I^ were found in either forward or reverse orientation. To introduce *Sma*I site next to the initiation codon of the *OsY37*^I^, pProm17 was amplified by PCR with UPSPE (located −884 bp upstream of the initiation codon) and PromRv (possessing *Sma*I site together with ATG initiation codon), then cloned in pGEM-T Easy vector to generate pProm17seg1 (Figure S6). Faithful amplification was verified by DNA sequencing. The fragment was excised from the clone pProm17seg1 by *Spe*I (in the promoter region) and *Sma*I, then ligated to the pProm17 formerly digested with *Spe*I (in the promoter region, 729 bp upstream of the initiation codon) and *Sma*I (in the vector region), generating the clone pProm17-10 (Figure S6). In this clone, *Spe*I/*Sal*I segment of Prom17 was replaced by *Spe*I/*Sma*I segment. pProm17-10 was digested with *Hin*dIII (in pUC19) and *Sma*I, and then ligated to pActSRDXG which had been digested with *Hin*dIII and *Sma*I, generating pProm17-10-1. In this clone, *OsY37*^I^ promoter was immediately followed by *Sma*I site and SRDX domain region. Afterward, full-length *OsY37*^N^ was amplified by PCR with primers OsyCRESTFw and OsyCRESTRv, both containing *Sma*I sites, and cloned in pGEM-T Easy vector, generating CR3. Accurate sequence of CR3 was determined. Further, coding region was excised from the CR3 by *Sma*I digestion, and then ligated to *Sma*I-digested pProm17-10-1. Right orientation of the coding region relative to the promoter was isolated, and termed pPromOsY37SRDX7 (Figure S6). In this clone, promoter is followed by coding sequence of *OsY37*^N^ fused with the SRDX domain. Both the plant vectors, pIG121Hm, and pPromOs37^N^SRDX7 were digested with *Hin*dIII and ligated, to obtain co-integrate clone pIG24, which was used to transform rice plants.

### 4.8. Nitrogen Starvation

Rice seeds with intact coats were surface-sterilized with 1.5% pefurazoate (Hokko Chemical Industry Co., Tokyo, Japan) for 10 min, thoroughly washed with water and imbibed for 48 h. The seeds were sown on soil containing 0.6 g nitrogen, 0.8 g phosphorous, and 0.5 g potassium sources, then were allowed to grow in a greenhouse until the seedlings attained about 5 cm in height. Single seedlings were transferred to 250 mL vermiculite in 300 mL pots containing 100 mL tap. Two hypotonic solutions (20 mL) were used; one contained a nitrogen source (N-medium) while the other was devoid of a nitrogen source (N-free medium) for nitrogen starvation. The N-medium was composed of 1 mM (NH_4_)_2_SO_4_, 15 mM KH_2_PO4, 20 mM KNO_3_, and 39 mM NH_4_NO_3_, as major nutrient sources; 0.5 mM MgCl_2_, 0.5 mM CaCl_2_, 2mM FeNa (EDTA), and 5 mM citric acid as minor sources; and 16 µM H_3_BO_4_, 22 µM MnSO_4_, 37 µM ZnCl_2_, 5.9 µM CuCl_2_, 0.8 µM (NH_4_)_6_Mo_7_O_24_, and 4.1 µM CoCl_2_ as sources of trace elements. For the N-free medium, N sources, (NH_4_)_2_SO_4_, KNO_3_, and NH_4_NO_3_ were replaced by 20 mM K_2_SO_4_. Hence seedlings were incubated in a growth chamber under constant light (300 µmol m^−2^ s^−1^) at 28 °C. The hypotonic nutrient solution was refreshed every 2 weeks.

### 4.9. RT-PCR

Total RNA (6 µg) was extracted using RNeasy Plant Mini Kit (QIAGEN), then treated with RNase-free DNase I (RQ1, 5 U; Promega, Madison, WI, USA) in 40 mM Tris-HCl (pH 8.0), 10 mM MgSO_4_, and 1 mM CaCl_2_ for 30 min at 37 °C. RQ1 was inactivated by phenol treatment, and the RNA was precipitated with ethanol. cDNA was synthesized from the RNA (1 µg) by reverse transcriptase using the Transcriptor First Strand cDNA Synthesis Kit (Roche, Indianapolis, IN, USA) in the presence of an RNase inhibitor (Rnasin Plus RNase Inhibitor; Promega). The enzymes were heat inactivated at 85 °C for 5 min. Aliquots were withdrawn and amplified by PCR using *Taq* DNA polymerase (Titanium DNA Polymerase; Clontech) with the following protocol: 94 °C for 30 s (1 cycle), then 94 °C for 30 s, 60 °C for 30 s, and 72 °C for 1 min (30 cycles), and finally 72 °C for 7 min (one cycle). The primers used to amplify *OsY37* were the forward primer osy-2, which extends from 2 bp upstream of the initiation codon, and the reverse primer osy752 starting from 780 bp downstream of the initiation codon.

### 4.10. Transformation of Rice and Arabidopsis

*Japonica* rice *Oryza sativa* cv. “Nipponbare” and “Kinmaze” were transformed using the callus method [49]. Briefly, seeds were husked, surface-sterilized in 2% sodium hypochlorite for 25 min, then cultured on medium supplemented with 2,4-D (2,4-Dichlorophenoxyacetic acid, 1 µg mL^−1^) and allowed to form callus. The callus was infected with *A. tumefaciens* strain EHA101 carrying a chimeric plasmid clone in the presence of acetosyringone (10 µg mL^−1^) and selected for Hgr^R^ (50 µg mL^−1^) resistance. Subsequently, callus was transferred to regenerated to shoots on medium containing NAA (naphthalene acetic acid, 2 ng mL^−1^), kinetin (2 µg mL^−1^), and Hgr (50 µg mL^−1^). T1 generation of *OsY37*-overexpressing (OE) and *OsY37*-RNAi lines and primary transformants of *OsY37*-SRDX lines were subjected to further study.

Transformation of *Arabidopsis thaliana* ecotype Columbia was performed according to the floral dip method [54] by infection with *Agrobacterium tumefaciens* strain GV3101 carrying the plasmid pBC35SOsY37SRDXG or pBCActOsY37SRDXG. A total of 4000 T_1_ seeds were surface-sterilized in 1.0% (*w*/*v*; chlorine) sodium hypochlorite together with 0.02% (*w*/*v*) Triton X-100 for 7 min. The sterilized seeds were sown on MS medium [55] containing 30 µg mL^−1^ Hgr to obtain 40–100 transgenic seedlings.

### 4.11. Particle Bombardment

DNA was adsorbed to gold particles (1.0 µm diameter) in 125 µL solution containing 5 µg DNA, 3 mg gold particles, 1 M CaCl_2_, and 0.016 M spermidine for 3 min at room temperature. The DNA/gold particle complexes were recovered by centrifugation (7000× *g*, 10 s) and dissolved in ethanol. The entire mixture was placed onto a micro-carrier and air-dried. Onion bulb epidermal peels were placed on basal MSB5 agar plates, composed of MS salts [55] supplemented with Gamborg B5 vitamins [56], 3% (*w*/*v*) sucrose and 0.8% (*w*/*v*) BA10 agar (Ina Food, Nagano, Japan). DNA/gold particles were bombarded twice onto the surface of the onion epidermal cells at 1100 psi working with a 900 psi rupture disk under vacuum (28 in. Hg) using a Biolistic PDS-1000/He Particle Delivery System (Bio-RAD, Hercules, CA, USA). The plates were incubated for 16 h of dark condition at 30 °C. The presence of GFP protein was examined by fluorescence microscopy (BX41, OLYMPUS, Nishi-Shinjuku, Tokyo, Japan) under ultraviolet light irradiation with appropriate filters. To visualize nuclei, samples were stained with 10 mL of 1 µg mL^−1^ 4′,6-diamidino-2-phenylindole dihydrochloride (DAPI) dissolved in water for 10 min then viewed using a fluorescence microscope under ultraviolet light irradiation with appropriate filters.

## 5. Conclusions

We isolated a member of the NAC transcription factor family genes *OsY37*^I^ from *indica* and identified the gene to be an orthologue of *OsNAC011* from *japonica* rice. *OsNAC011* was renamed *OsY37*^N^. Our results show that *OsY37* is a senescence associated gene encoding a novel protein involved in plant growth and development; implicated in heading and flowering times, and presumably regulating the senescence process by promoting chlorophyll degradation in rice. We conclude that *OsY37* acts as a positive regulator of senescence, however further analysis is required to determine the specific pathway in which *OsY37* is involved.

## Figures and Tables

**Table 1:** List and sequence of primers.

Primer	Sequce
FwOsGFP	AGGAGGTGTACAAGATGAGCGGGATGAATTCGCTT
M13Rv	CAGGAAACAGCTATGAC
IndFw	TCGAGGAGGAGGGGGTGGGC
IndRv	GCAAATAATGTGAACTAGAATGGTAC
OsYIFw	ATGAGCGGGATGAATTCGCT
OsYIRv	ACTGAGTGAGTTCCACATTT
Fw24	TCGAGGAGGAGGGGGTGGGCATGA
Rv869	AGTTCCACATTTGTGTGAAGGCATGCTCAGCCAAGTAATCAAATCCATC
FwOsSR	ATGAGCGGGATGAATTCGCT
RvSRDX	ACTGAGTGAGTTCCACATTT
osy-2	GCATGAGCGGGATGAATTCG
osy752	GATCGCCCTACCCATATTGT
PromRv	GAATTCATCCGCTCATCCCGGGCCCCTCCTCCTCGAATTCCT
UPSPE	GGATTGTAAGATAAGTCCTC
OsyCRESTFw	GAATTCGAGGAGGAGGGGCCCGGGATGAGCGGGATGAATTCGCT
OsyCRESTRv	TATATATCTCCTTGGAAATTCCCGGGACTGAGTGAGTTCCACAT
hph1	ATGTCCTGCGGGTAAATAGC
hph2	CGTCTGCTGCTCCATACAAG
OsActinF318	GGTATCGTCAGCAACTGGGATGATATGG
OsActinR675	GCTCCGTCAGGATCTTCATGAGGTAATC
OsyPri1	TGCCCGGTGATGGTCGACGT
SRDX-R	TTAAGCGAAACCCAAACGGAGTTCTAG

## Supplementary Materials

Supplementary materials can be found at www.mdpi.com/1422-0067/18/10/2165/s1.

## Abbreviations

pAct	Rice actin promoter
pCaMV35S	Cauliflower mosaic virus 35S promoter
CRES-T	Chimeric repressor silencing technology
GFP	Green fluorescent protein
Hgr^R^	Hygromycin-resistant
MS	Murashige and Skoog
NAC	NAM, ATAF, and CUC
RNAi	RNA interference
