# Supplementary material for: The NAC Transcription Factor Gene OsY37 (ONAC011) Promotes Leaf Senescence and Accelerates Heading Time in Rice
Source: Int J Mol Sci. 2017 Oct 17;18(10):2165. doi: 10.3390/ijms18102165 (PMC5666846; doi:10.3390/ijms18102165)
Supplement: Supplementary file 1 [file ijms-18-02165-s001.pdf]

The NAC Transcription Factor Gene *OsY37* (ONAC011) Promotes Leaf Senescence and Accelerates Heading Time in Rice

Yousra El Mannai, Kenta Akabane, Keiichiro Hiratsu, Namiko Satoh-Nagasawa and Hiroetsu Wabiko

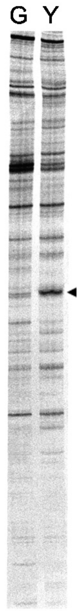

Figure S1. Differential display of gene expression profiles of young green leaves and senescing leaves. Healthy green leaves were excised from rice "Akitakomachi" plants 10 days before heading (denoted as G) and senescing yellow leaves (Y) at 34 days after heading. Total RNA was extracted and subjected to cDNA synthesis followed by DNA amplification with [α-^{35}S]-dATP using a forward primer (CAGGCCCTTC) and reverse primer (oligo-dT). DNA was separated by 5% polyacrylamide gel electrophoresis and identified by autoradiography. The arrowhead indicates the band preferentially amplified in yellowing leaves, corresponding to the *OsY37* gene segment.

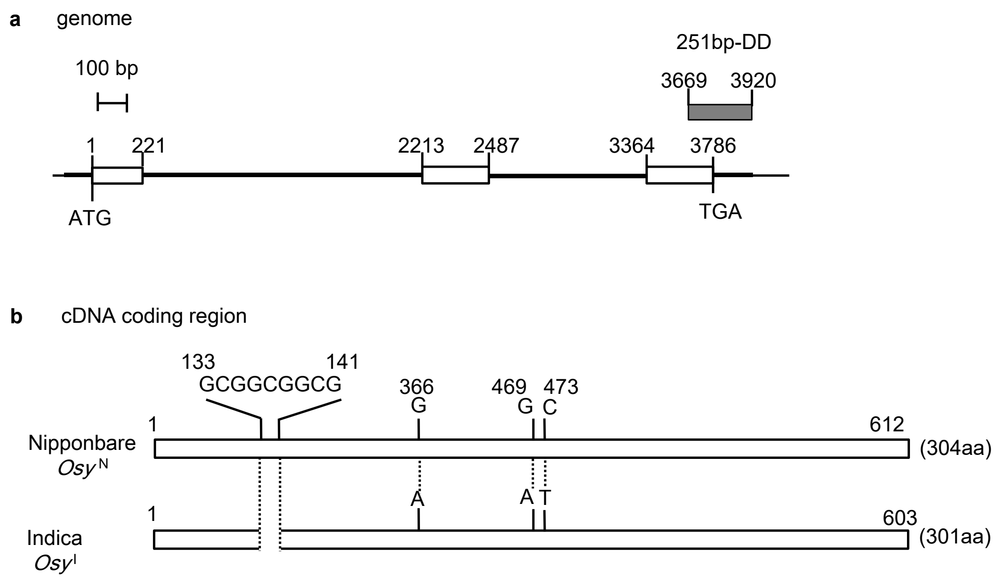

Figure S2. Organization of the *OsY37* gene. (**a**): Genome segment including the *OsY37* gene. Solid thick line indicates the full-length of the transcripts including introns. Solid thin line shows DNA segment outside the transcripts. Open bars denote exon regions. Numbering in the genome region starts at the first nucleotide of the initiation codon, ends at the third position of the termination codon, and denotes the positions of the intron/exon configuration. Dark gray box indicates the segment obtained by the differential display. (**b**): Open bars show the coding region *OsY37*^{N} (*japonica,* Nipponbare) and *OsY37*^{I} (*indica,* IR36). Numbers are based on the coding sequence, starting at the initiation codon. Numbers with vertical bars above the open bars show the nucleotide positions different between *OsY37*^{N} and *OsY37*^{I}. Nucleotide residues 133-141 in *OsY37*^{N} are apparently deleted in *OsY37*^{I}. Number of the amino acid (aa) residues are also shown.

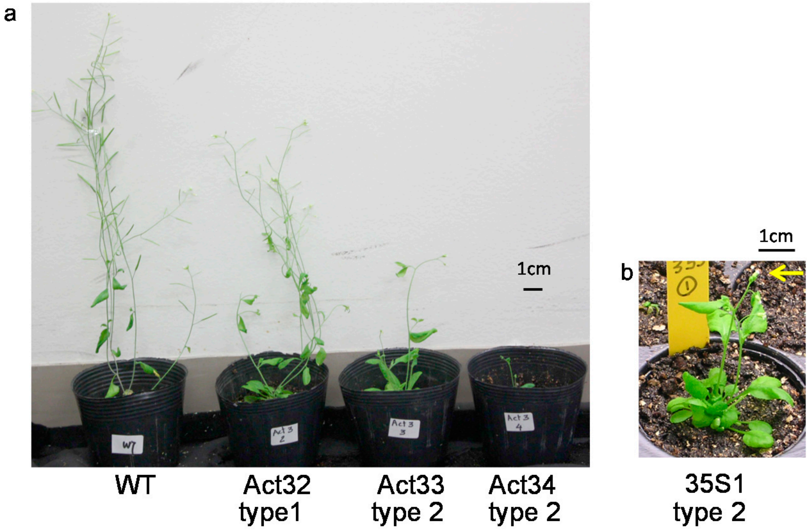

Figure S3. Phenotypic alteration of *Arabidopsis thaliana* transgenic for the *OsY37*^{I} gene fused to the SRDX sequence. Seeds of T1 generation carrying the transgenes pAct::*OsY37*^{I}/SRDX and p35S::*OsY37*^{I}/SRDX were sown on selective MS medium containing 30 μg/ml hygromycin. After 19 days, Hgr^{R} seedlings were transferred to soil and allowed to grow for 26 days to maturity. Transgenic lines possessing pAct::*OsY37*^{I}/SRDX (**a**) and p35S::*OsY37*^{I}/SRDX (**b**). The yellow arrow in (**a**) denotes a terminal flower.

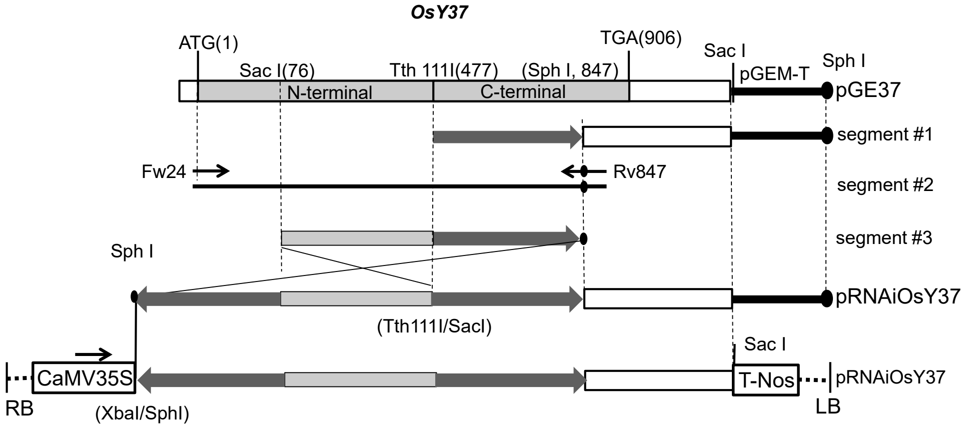

Figure S4. Construction of RNAi-*OsY37*^I in the plant vector. Full-length cDNA of *OsY37*^I clone, pGE37 is shown. Gray open bar of pGE37 indicates the N-terminal half region corresponding to the conserved NAC domain, and the unique C-terminal half region. Solid bars denote the pGEM-T vector. Nucleotide positions shown in parentheses are numbered, starting at the initiation codon. Ovoid dots indicate *Sph*I digestion sites. The major part of NAC domain was excised to generate the segment #1. Fw24 and Rv869 primers were used to amplify pGE37 to generate the segment #2. The segment #2 was further cloned to segment #1 to obtain pRNAiOsY37. The segment corresponding to RNAi was further cloned into the plant vector pIG121Hm to generate the final clone, pIGRNAiOsY37. RB and LB that flank RNAi together with higromycin and kanamycin resistance genes (omitted in this figure) will be excised and transferred to plant chromosome.

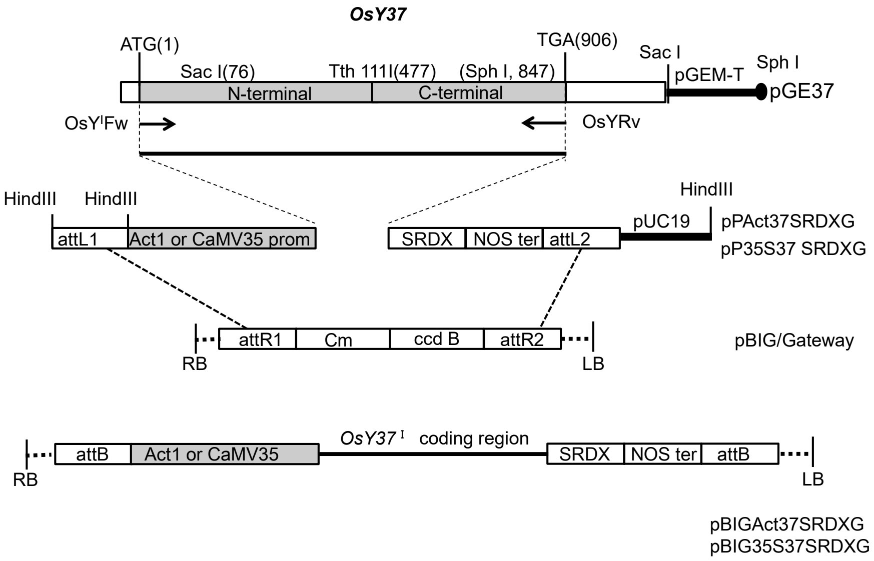

Figure S5. OsY37^I-SRDX fusion polypeptide construct under the control of Actin and CaMV35S promoters. Solid line below pGE37 indicates the coding region of *OsY37*^I segment amplified with OsY ^IFw and OsY ^IRv primers. pPActSRDXG and p35SSRDXG contains the OsY37^I fused with SRDX polypeptide segment under the control of Act1 and CaMV35S promoters respectively. A DNA cassette for the Gateway Vector Conversion System had been inserted at *Hin*dIII site of pBIG binary plant vector [40] to generate pBIG-Hgr. Thick dotted lines connect the recombination portion in the Gateway system to introduce the promoter: *OsY37*^I construct into the plant vector to generate pBIGAct37SRDX and pBIG35S37SRDX. Final constructs confer Hgr^R on plants.

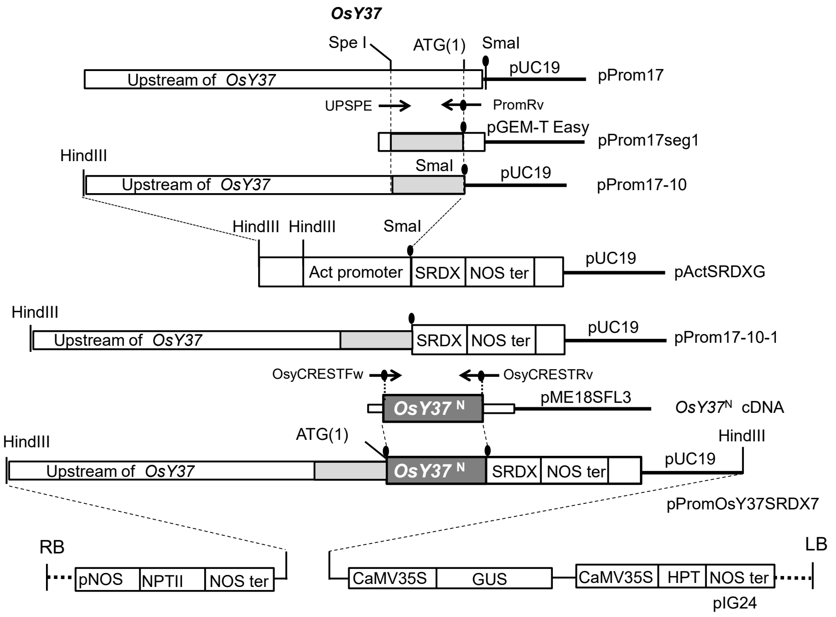

Figure S6. Diagram to generate *OsY37* promoter: *OsY37*^{N}-SRDX version into the plant vector. Names of the clones are underlined. Open bars indicate the cloned recombinant segments including the promoter, full-length coding region of *OsY37*^{N}, and SRDX sequence. Solid lines denote vector regions and are not shown to scale. The open pale gray bar shows the portion upstream of the *OsY37*^{N} surrounding the initiation codon and was replaced by the segment amplified by primers UPSPE and RromRV to generate Prom17-10. The promoter region in Prom17-10 was fused to SRDX sequence to create Prom17-10-1. The coding region of *OsY37*^{N} was amplified by primers OsyCRESTFw and OsyCRESTRv and inserted in front of SRDX to generated PromOsY37SRDX7. Co-integrate between PromOsY37SRDX7 and pIG121Hm vector was made to create pIG24. T-DNA segment flanked by LB and RB is transferred to plant cells. NPTII and HPT genes confer kanamycin and hyromycin resistance in plants.
